# Cleistanthins A and B Ameliorate Testosterone-Induced Benign Prostatic Hyperplasia in Castrated Rats by Regulating Apoptosis and Cell Differentiation

**DOI:** 10.7759/cureus.32141

**Published:** 2022-12-02

**Authors:** Santosh Kumar S C, Raveendran R, Rajesh Nachiappa Ganesh

**Affiliations:** 1 Pharmacology, Jawaharlal Institute of Postgraduate Medical Education & Research, Puducherry, IND; 2 Pathology, Jawaharlal Institute of Postgraduate Medical Education & Research, Puducherry, IND

**Keywords:** apoptosis, dihydrotestosterone, androgen receptor, proliferating cell nuclear antigen, cyclin d1, caspase-3, il-6, stat-3, cleistanthus collinus

## Abstract

Background

The aging male population is at higher risk for benign prostatic hyperplasia (BPH) wherein increased proliferation of stromal and epithelial cells of the prostate is observed. In this study, we investigated the effect of cleistanthins A and B on the inhibition of testosterone-induced BPH in castrated rats.

Methodology

Male Wistar rats were divided into eight groups (n = 6) and surgical castration was performed. BPH was induced by the administration of testosterone propionate in corn oil at 5 mg/kg for four weeks. The control group received corn oil, and the model group received testosterone propionate. The standard treatment group received finasteride orally along with testosterone. Cleistanthins A and B at 0.3, 1, and 3 mg/kg were administered by oral gavage along with testosterone. After four weeks, rats were sacrificed, and prostates were weighed and assessed for histomorphological, inflammatory, apoptotic, and proliferative markers.

Results

Cleistanthins A and B decreased prostatic enlargement and histopathological abnormalities. Elevated serum dihydrotestosterone levels were lowered significantly in both the cleistanthin A and cleistanthin B groups compared to the BPH model group. Cleistanthins A and B significantly lowered the serum interleukin (IL)-1β and tumor necrosis factor-alpha inflammatory markers in the test groups. Western blot analysis revealed cleistanthin A downregulated the IL-6, signal transducer and activator of transcription 3/cyclin D1 signaling pathway. Both cleistanthins A and B upregulated the apoptotic markers caspase-3 and cleaved caspase-3, whereas the cell proliferation markers cyclin D1 and proliferating cell nuclear antigen were found to be downregulated.

Conclusions

Both cleistanthins A and B inhibited BPH in a rat model by apoptotic induction and impeded cell proliferation.

## Introduction

Benign prostatic hyperplasia (BPH) is a common chronic non-malignant debilitating disease condition in the geriatric male population which severely affects the quality of life. Autopsy data suggest that men aged 50-60 years have a histological prevalence of 50% and those aged 80 years and above have a prevalence of 90% [1]. The pathophysiology of BPH is not known clearly, and factors such as aging, hormonal imbalance, androgens, epithelial-mesenchymal interactions, and estrogens are involved in the pathogenesis of BPH [2]. The rapid proliferation of stromal smooth muscle cells, epithelial cells, and connective tissue of the prostate is linked to hyperplasia of the prostate gland [3]. An increase in prostatic volume (static) and smooth muscle tone (dynamic) are involved in the pathology of BPH. The static component physically compresses the urethra and the outlet of the bladder, and the dynamic component increases the prostatic smooth muscle tension [4]. The growth and development of the prostate gland are mediated by androgens such as testosterone and dihydrotestosterone (DHT) [5,6].

5-α reductase inhibitors (5-ARI), namely, finasteride and dutasteride, act on the static component and decrease the prostate size, whereas α-adrenergic receptor antagonists, namely, tamsulosin and alfuzosin, act on the dynamic component and decrease the prostatic smooth muscle tone [7]. However, both 5-ARIs and α-adrenergic receptor antagonists produce undesirable side effects which include loss of libido, erectile dysfunction, ejaculatory disorders, orthostatic hypotension, and dizziness [8,9]. The side effects and the resistance to existing pharmacotherapy of BPH mandate the search for novel drug molecules.

Cleistanthins A and B are cytotoxic aryl naphthalene lignan glycosides commonly found in the *Cleistanthus collinus* plant. Despite its toxicity, cleistanthin A possesses anticancer and α-adrenergic antagonistic properties [10,11]. A study by Pradheepkumar et al. suggested that the cytotoxic effect of cleistanthin A in cervical carcinoma (Si Ha) and p53 deficient (K562) cell lines is due to DNA damage, apoptosis, and inhibition of DNA synthesis [12]. CA is a potent V-ATPase proton pump inhibitor, and different types of cancer cells overexpress V-ATPases, which is correlated positively with the metastasis and invasion of tumor cells [13,14]. Cleistanthin B decreases cell viability, increases DNA strand breaks, and induces chromatid and isochromatid breaks and gaps in Chinese hamster ovary cells [15]. Cleistanthin B exerts in vivo anti-tumor effect in Ehrlich’s ascites carcinoma and Dalton’s ascites lymphoma cell line tumor-bearing animals by significantly increasing overall survival [16].

Parasuraman et al., Priyadharsini et al., and Sahoo et al. have shown that cleistanthins A and B have prominent α-adrenergic antagonistic action on the peripheral vascular system [11,17,18]. Cytotoxic and anti-proliferative herbal-based formulations and plant-derived compounds have been investigated for the treatment of BPH, and preclinical studies on these drugs were found to be effective [19]. With this background, this study aimed to evaluate the efficacy of cleistanthins A and B in testosterone-induced BPH in orchiectomized rats.

## Materials and methods

Animals

Male Wister rats weighing 200 ± 50 g were procured from the Central Animal House, Jawaharlal Institute of Postgraduate Medical Education & Research (JIPMER), Puducherry. The rats were maintained under controlled environmental conditions throughout the experiments with 12-hour light and dark cycles alternatively and were acclimatized to departmental laboratory conditions for a week before the start of the experiments. The experiments were conducted in accordance with the guidelines established by the Committee for the Purpose of Control and Supervision of Experiments on Animals (CPCSEA). The study was approved by the Institute Animal Ethics Committee (IAEC).

Chemicals and drugs

Testosterone propionate (TP) (catalog number: 86541, lot number: BCBP3129V), finasteride (catalog number: 34202, lot number: SZBF337XV), and corn oil (catalog number: C8267, lot number: MKBS6944V) were purchased from Sigma-Aldrich. Cleistanthins A and B were isolated from the leaves of *Cleistanthus collinus*, as described previously [11,20], and the structure elucidation was performed by ^1^H, ^13^C, and Fourier-transform infrared spectroscopy. TP was dissolved in corn oil, whereas finasteride, cleistanthin A, and cleistanthin B were dissolved in 0.5 % carboxymethyl cellulose.

Experimental benign prostatic hyperplasia and treatments

Surgical castration was performed on rats to avoid the influence of endogenously produced testosterone. Ketamine (70 mg/kg) administered via the intraperitoneal route was used as an anesthetic agent during surgical castration. Compression of the lower abdomen exposed the rat testes, following which a midline vertical incision was made on the scrotal sac and slight traction was applied to deliver the testes out of the incision. The spermatic cord and the blood vessels near the upper pole of the testes were ligated with chromic catgut 2-0 and the testes were resected out beyond the ligature. Finally, the scrotal sac was sutured by a simple suture technique using chromic catgut 2-0 on a 30 mm half-circle round-bodied needle. The rats were allowed to recover from surgical castration for 10 days and were provided with food and water ad libitum.

After recovery, BPH was induced in castrated rats by subcutaneous (SC) administration of TP (5 mg/kg) in corn oil for 28 days. Rats were randomly divided into the following nine groups (n = 6): group 1 (the control group) was administered with corn oil SC; group 2 (the BPH model group) received TP in corn oil SC; group 3 (the standard treatment group) received finasteride (1 mg/kg) orally along with TP by the SC route; and groups 4, 5, 6, 7, 8, and 9 received test drugs cleistanthin A and cleistanthin B orally at 0.3 mg/kg, 1 mg/kg, and 3 mg/kg body weight, respectively, along with TP by SC for 28 days (Table 1).

**Table 1:** Experimental groups and treatments.

Groups (n = 6)	Treatment
Negative control	Corn oil 1 mL/kg/day subcutaneous for 28 days
Model	Testosterone 5 mg/kg/day subcutaneous in corn oil for 28 days
Finasteride	Testosterone 5 mg/kg/day subcutaneous in corn oil + finasteride 1 mg/kg/day p.o. for 28 days
Cleistanthin A 0.3 mg	Testosterone 5 mg/kg/day p.o. in corn oil + cleistanthin A 0.3 mg/kg/day p.o. for 28 days
Cleistanthin A 1 mg	Testosterone 5 mg/kg/day p.o. in corn oil + cleistanthin A 1 mg/kg/day p.o. for 28 days
Cleistanthin A 3 mg	Testosterone 5 mg/kg/day p.o. in corn oil + cleistanthin A 1 mg/kg/day p.o. for 28 days
Cleistanthin B 0.3 mg	Testosterone 5 mg/kg/day p.o. in corn oil + cleistanthin B 0.3 mg/kg/day p.o. for 28 days
Cleistanthin B 1 mg	Testosterone 5 mg/kg/day p.o. in corn oil + cleistanthin B 1 mg/kg/day p.o. for 28 days
Cleistanthin B 3 mg	Testosterone 5 mg/kg/day p.o. in corn oil + cleistanthin B 3 mg/kg/day p.o. for 28 days

Prostatic index, percentage of inhibition, blood sampling, and prostate tissue collections

The prostatic index and percentage of inhibition were calculated based on a previous study [21]. After the completion of the experiments, the rats were fasted for 12 hours, and on the 29th day, up to 2-3 mL of blood was drawn by cardiac puncture. The serum was separated and stored in a -20°C freezer for further analysis. The prostate gland was dissected by making a midline incision in the pelvic region to expose the urinary bladder. The prostate lobes identified at the base of the urinary bladder were resected and placed in 0.9% saline. The prostate gland was weighed and the prostatic index and the percentage of inhibition of growth of the prostate were calculated, as described.

Histopathological examination of prostate tissue

Prostate gland sections were fixed with 10% neutral buffered formalin and treated with a series of graded alcohol to dehydrate and embedded in paraffin. Subsequently, 4 µM thick sections were cut using a microtome and stained with hematoxylin and eosin (H&E) for histopathological examination

Determination of serum dihydrotestosterone concentration by enzyme-linked immunosorbent assay

The DHT concentration of serum was measured by following the user manual instructions for the rat DHT enzyme-linked immunosorbent assay (ELISA) kit (catalog number: E0563Ra, lot number: E170419-6, Bioassay Technology Laboratory., Shanghai, China). The optical density (OD) was determined using a 680XR Bio-Rad microplate reader set at 450 nm.

Determination of serum pro-inflammatory markers by enzyme-linked immunosorbent assay

Serum inflammatory markers, interleukin-1β (IL-1β) (catalog number: ELR - IL-β, lot number: 0113170721) and tumor necrosis factor-alpha (TNF-α) (catalog number: ELR - TNF-α, lot number: 0113170709) were measured by a 680XR Bio-Rad microplate reader with the OD set at 450 nm, as per the manual instructions provided by the rat ELISA kit (Ray-biotech).

Western blot analysis

Snap-frozen prostate tissues stored at -80°C were immediately placed on ice and weighed. Subsequently, 1,000 µL of radioimmunoprecipitation assay (RIPA) buffer (R0278, Sigma-Aldrich) was added to 50 mg of prostate tissue along with a protease inhibitor cocktail (P9599, Sigma-Aldrich) and homogenized in a hand-held glass mortar and pestle on ice. The homogenized prostate tissues were centrifuged at 12,000 rpm for 20 minutes, and the supernatant was subjected to Bradford assay (B6916, Sigma-Aldrich) for protein quantification. Further, 30 µg of protein aliquots were separated on sodium dodecyl sulfate-polyacrylamide (SDS-PAGE) with 8-12% gels. The resolved proteins were transferred to a nitrocellulose membrane and washed with tris-buffered saline with Tween 20 (TBST) for five minutes.

The nitrocellulose membranes were incubated with blocking solution (5% bovine serum albumin) for one hour and washed with TBST for five minutes. The blots were then incubated with primary antibodies for proliferating cell nuclear antigen (PCNA) (CST-2586), cyclin D1 (CST-2978S), caspase-3 (CST-96626), cleaved caspase-3 (CST-9661S), IL-6 (CSB-PA005546/H0228Y), signal transducer and activator of transcription (STAT-3) (CSB-PA004173/H0228Y), beta-actin (E-AB-20058/AF0501), and androgen receptor (SC-7305) overnight at 40°C. The blots were washed with TBST thrice for eight minutes and incubated with secondary immunoglobulin G (IgG) anti-rabbit antibody (SC-2004) for one hour. The immunoreactive bands were visualized with the use of an enhanced chemiluminescence solution.

Statistical analysis

The study results are presented as mean ± standard error of the mean (SEM). Multiple comparison analysis was performed using one-way analysis of variance (ANOVA) followed by the post-hoc Tukey-Kramer test. Statistical analyses were conducted using GraphPad Instat version 3.

## Results

Cleistanthins A and B inhibit prostatic hyperplasia induced by testosterone in castrated rats

The mean prostate weight of the negative control group was 0.31 ± 0.03 g, and the administration of TP for 28 days to the model group increased the prostate weight to 0.74 ± 0.03 g, whereas the mean prostate weight of the rats treated with finasteride was 0.54 ± 0.06 g. When cleistanthin A at doses of 0.3 mg, 1 mg, and 3 mg was administered along with testosterone for 28 days to the castrated rats, the mean prostate weights were 0.73 ± 0.04 g, 0.59 ± 0.05 g, and 0.55 ± 0.04 g, respectively, and with cleistanthin B at similar doses, the mean prostate weights were 0.74 ± 0.04 g, 0.61 ± 0.04 g, and 0.56 ± 0.04 g, respectively, as shown in Table 2.

**Table 2:** Effect of cleistanthins A and B on rat prostate weight, prostatic index, and percentage of inhibition. Data are presented as mean ± SEM, and statistical analysis was performed using one-way ANOVA followed by the Tukey-Kramer’s test. *** p < 0.001, ** p < 0.01 and * p < 0.05 when compared to negative control group. ## p < 0.01 and # p < 0.05 when compared to the model group. NC = negative control; M = model group (testosterone propionate); F = finasteride group; CA = cleistanthin A; CB = cleistanthin B; SEM = standard error of the mean; ANOVA = analysis of variance

Groups (n = 6/group)	Body weight (g) (Mean ± SEM)	Prostate weight (g) (Mean ± SEM)	Prostatic index (Mean ± SEM)	% of inhibition
NC (corn oil)	224.66 ± 9.83	0.31 ± 0.03	1.38 ± 0.14	
M (5 mg/kg)	246.83 ± 7.18	0.74 ± 0.03 ***	3.01 ± 0.07 ***	
F (1 mg/kg)	239.66 ± 8.76	0.54 ± 0.06 *	2.23 ± 0.20 **^##^	46.51
CA (0.3 mg/kg)	247.33 ± 8.67	0.73 ± 0.04 ***	2.98 ± 0.12 ***	2.53
CA (1 mg/kg)	242.30 ± 5.79	0.59 ± 0.05 **	2.42 ± 0.15 ***	35.18
CA (3 mg/kg)	238.83 ± 5.12	0.55 ± 0.04 *	2.31 ± 0.17 ***^#^	44.13
CB (0.3 mg)	246.83 ± 6.04	0.74 ± 0.03 ***	3.00 ± 0.08 ***	4.65
CB (1 mg)	252.66 ± 6.12	0.61 ± 0.04 ***	2.40 ± 0.14 ***	30.23
CB (3 mg)	246.83 ± 8.26	0.56 ± 0.04 **	2.27 ± 0.12 **^#^	41.86

The prostatic index of 3.01 ± 0.07 in the model group was significantly higher compared to 1.38 ± 0.14 in the negative control group and 2.23 ± 0.20 in the rats administered finasteride. The same was found to be 2.98 ± 0.12, 2.42 ± 0.15, and 2.31 ± 0.17 with the administration of cleistanthin A at 0.3 mg/kg, 1 mg/kg, and 3 mg/kg to BPH rats, respectively. When treated with cleistanthin B at doses 0.3 mg/kg, 1 mg/kg, and 3 mg/kg, the prostatic indices were 3.00 ± 0.08, 2.40 ± 0.14, and 2.27 ± 0.12, respectively.

Effect of cleistanthins A and B on the histomorphology of the prostate gland

**Figure 1:**
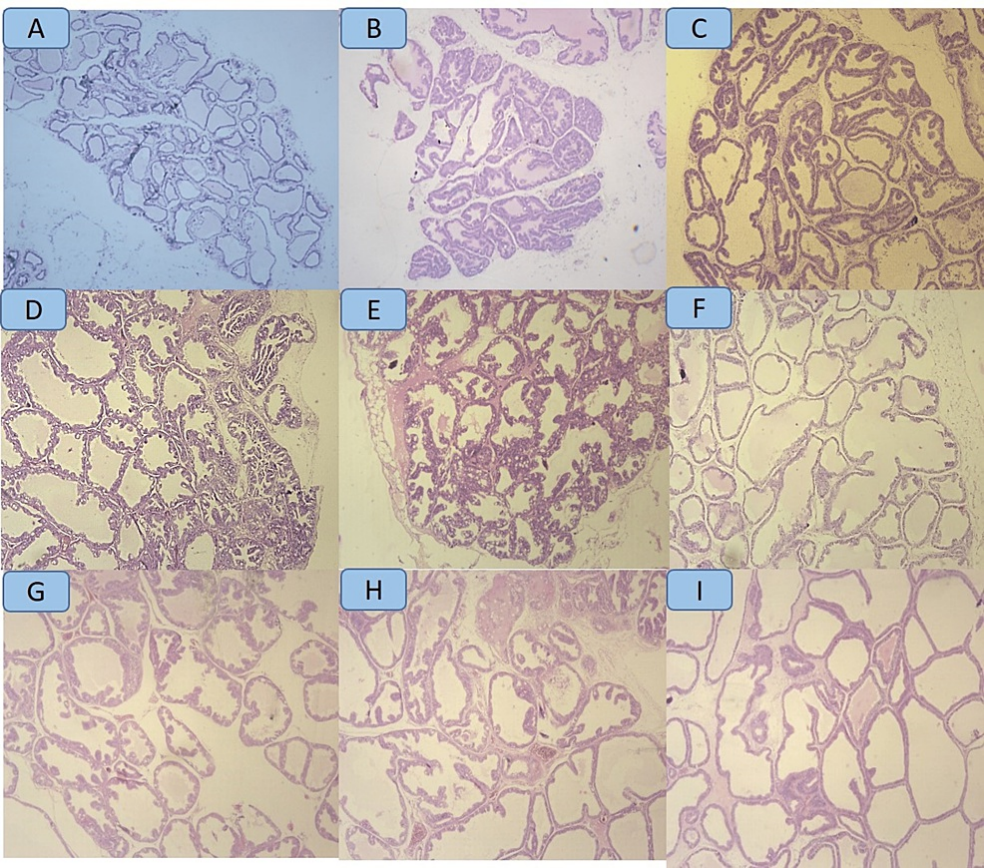
Histopathological analyses of the prostate sections of negative control (NC), model (M), finasteride (F), cleistanthin A (CA), and cleistanthin B (CB) groups. Hematoxylin and eosin stain at 200× magnification. A: The negative control group shows cystically dilated ducts lined by flattened low cuboidal epithelium. Stroma is loose and edematous. B: The model group shows significant papillary hyperplasia of the tall columnar lining ductal epithelium with multilayering of the cells. There is little intervening space between the ducts with dense stroma. C: The finasteride group shows cystically dilated ducts still highlighting focal papillary hyperplasia of the lining epithelium. The lining epithelium is focally lined by thinned-out cuboidal epithelium and focally by tall columnar epithelium. Intervening stroma is dense. D: The CA 0.3 mg group shows mild amelioration of the hyperplastic changes of the tall columnar lining epithelium with multilayering of the cells and dense prostatic stroma. E: The CA 1 mg group shows significant papillary hyperplasia of the tall columnar lining epithelium with dense prostatic stroma. F: The CA 3 mg group shows significant amelioration of the hyperplastic changes in the low cuboidal ductal epithelial lining with prominent cystic change. Stroma is inconspicuous. G: The CB 0.3 mg group shows cystically dilated ducts with focal papillary hyperplasia. Lining shows focal thinning with low cuboidal epithelium. H: The CB 1 mg group shows prominent cystically dilated ducts with focal papillary change and markedly flattened low cuboidal epithelium. I: The CB 3 mg group shows marked cystic dilatation of the ducts with near-total amelioration of the papillary change, and the lining is predominantly low cuboidal with edematous stroma.

In the negative control group, histological analyses of the prostate gland revealed that the epithelial cells were bilayered with flattened low cuboidal epithelium with no evidence of nuclear pleomorphism or prominence of nucleoli. The BPH model showed significant hyperplasia of the ductal epithelial lining with hypertrophy of the stroma in the periurethral portion of the prostate gland. The epithelial cells were multilayered with an increase in epithelial thickness and the cells in the form of papillary projections protrude into the luminal space. However, the rats treated with finasteride and cleistanthins A and B showed a significant amelioration of the hyperplastic changes in the prostate (Figure 1).

Effect of cleistanthins A and B on serum dihydrotestosterone levels

**Figure 2:**
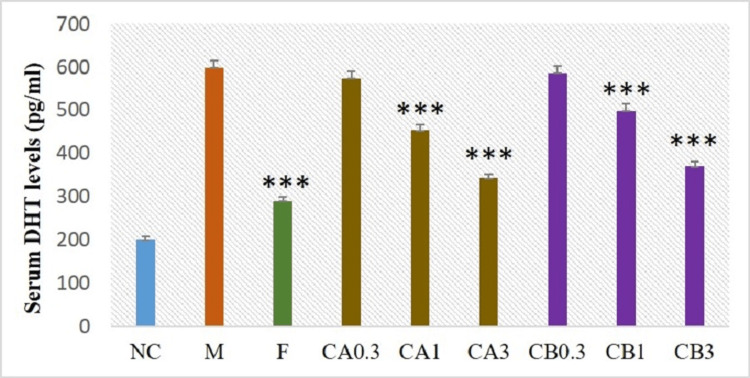
Effect of cleistanthins A and B on serum DHT levels of the BPH rat model. DHT levels were elevated in the testosterone-induced BPH model group. Treatment with finasteride, cleistanthin A, and cleistanthin B at 1 mg/kg and 3 mg/kg body weight significantly decreased the elevated serum DHT levels. Negative control (NC) group, model (M) group, finasteride (F) group, cleistanthin A (CA) group, and cleistanthin B (CB) group. Data are shown as mean ± SEM (n = 6). ***p<0.001 when compared to the model group (one-way ANOVA followed by the Tukey-Kramer test). DHT = dihydrotestosterone; BPH = benign prostatic hyperplasia; SEM = standard error of the mean; ANOVA = analysis of variance

The model group which received TP showed significantly higher levels of serum DHT (598.33 ± 17.55 pg/mL) when compared to the negative control group (201.16 ± 7.17 pg/mL) which received only the vehicle. Administration of finasteride and cleistanthins A and B at 1 and 3 mg doses markedly reduced the serum DHT levels (p < 0.005) (Figure 2).

Effect of cleistanthins A and B on inflammatory markers

**Figure 3:**
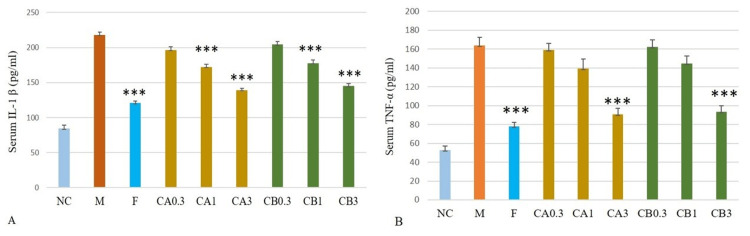
Effect of cleistanthin A and cleistanthin B on serum IL-1β (3A) and TNF-α (3B) levels of the BPH rat model. Both levels were elevated in the testosterone-induced BPH model group. Treatment with finasteride, cleistanthin A, and cleistanthin B at 1 mg/kg and 3 mg/kg body weight significantly decreased the elevated serum IL-1β and TNF-α levels. Negative control (NC), model (M) group, finasteride (F) group, and cleistanthin B (CB) group. Data are shown as mean ± SEM (n = 6). statistical analysis was performed using ANOVA followed by the Tukey-Kramer multiple comparisons test. *** significant difference compared to the model group (p < 0.001). IL-1β = interleukin-1β; TNF-α = tumor necrosis factor-alpha; BPH = benign prostatic hyperplasia; SEM = standard error of the mean; ANOVA = analysis of variance

The model group showed markedly higher levels of IL-1β and TNFα compared to the negative control group. With the administration of finasteride and cleistanthins A and B, the levels of inflammatory markers were reduced significantly, as shown in Figure 3.

Cleistanthin A downregulates the IL-6/STAT-3/Cyclin D1 signaling pathway and upregulates apoptotic machinery

**Figure 4:**
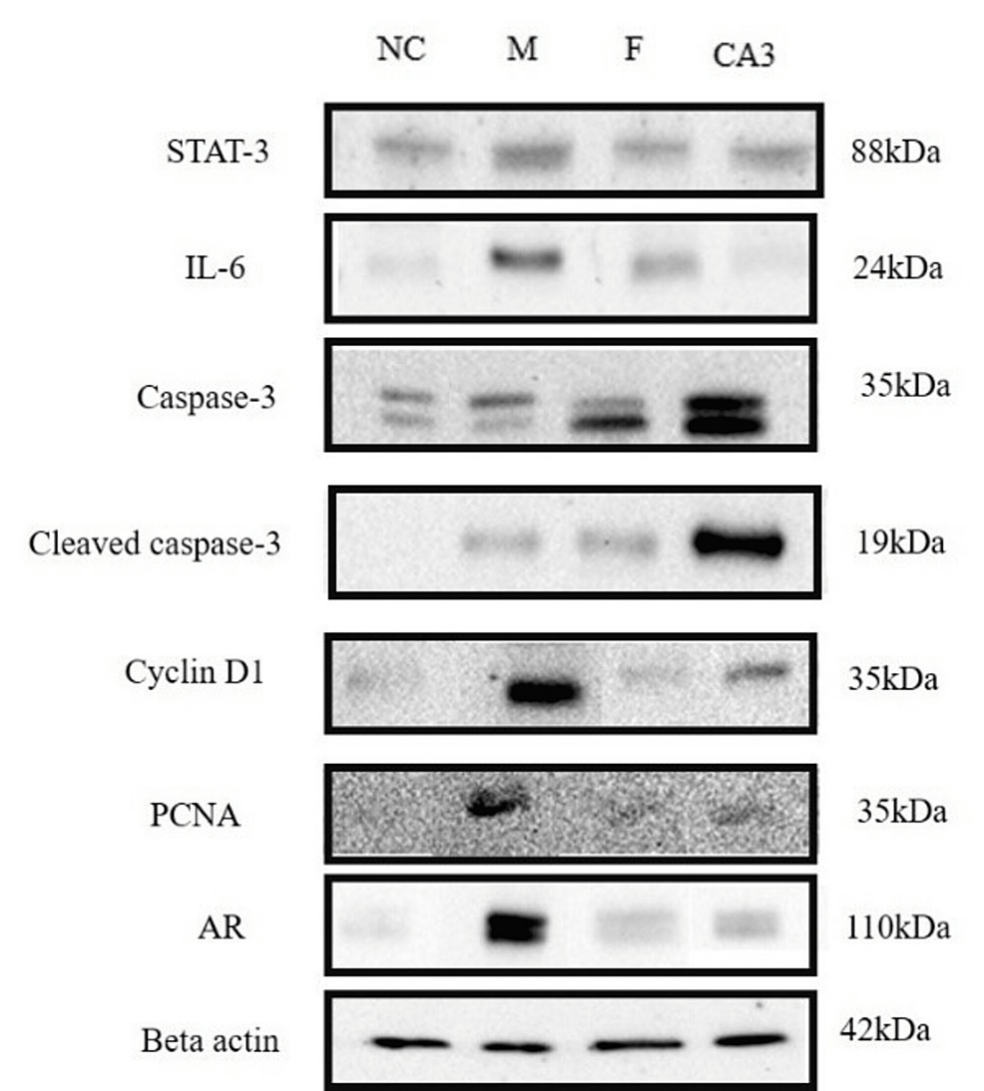
Western blotting for STAT-3, IL-6, caspase-3, cleaved caspase-3, cyclin D1, PCNA, and AR protein expressions in the prostate tissue of negative control (NC), model (M), finasteride (F), and cleistanthin A (3 mg/kg body weight) groups. STAT-3, cyclin D1, PCNA, and AR expressions were elevated significantly in the testosterone-induced BPH model, while treatment with cleistanthin A at 3 mg/kg body hindered these elevations. Apoptotic markers caspase-3 and cleaved caspase-3 protein expressions were downregulated in the BPH model group, and administration of cleistanthin A upregulated the expression of these apoptotic markers. β-actin was used as an internal control. STAT-3 = signal transducer and activator of transcription; IL = interleukin; PCNA = proliferating cell nuclear antigen; AR = androgen receptor; BPH = benign prostatic hyperplasia

Western blotting results showed an increased expression of STAT-3, IL-6, cyclin D1, and PCNA in the model group compared to the negative control group (Figure 4).

**Figure 5:**
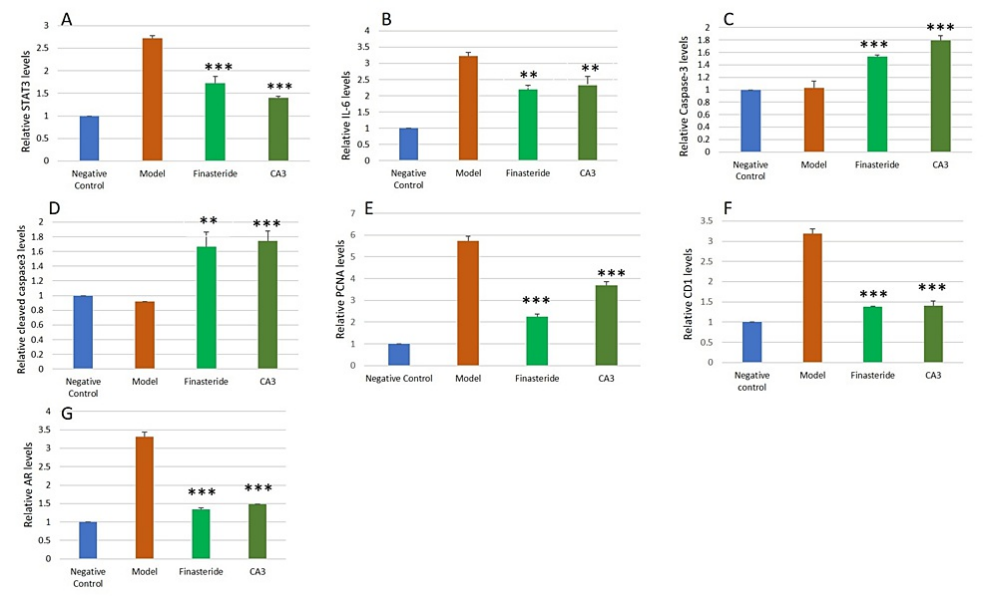
Relative STAT-3 (A), IL-6 (B), caspase-3 (C), cleaved caspase-3 (D), PCNA (E), cyclin D1 (F), and AR (G) protein levels of the negative control, model, finasteride, and cleistanthin A (3 mg/kg body weight) groups. β-actin was used as an internal control. Densitometric analysis was performed using Image Lab 6.0.1 software, Bio-Rad Laboratories. Data are shown as mean ± SEM (n = 6). Statistical analysis was performed using ANOVA followed by the Tukey-Kramer multiple comparisons test. ** significant difference compared to the model group (p < 0.01). *** significant difference compared to the model group (p < 0.001). STAT-3 = signal transducer and activator of transcription; IL = interleukin; PCNA = proliferating cell nuclear antigen; AR = androgen receptor; SEM = standard error of the mean; ANOVA = analysis of variance

Administration of finasteride and cleistanthin A at 3 mg/kg markedly reduced the relative expression of these proteins (Figures 5A-5D). Apoptotic markers such as caspase-3 and cleaved caspase-3 were not activated in the negative control and model groups, as observed with the immunoblotting. Finasteride treatment in the testosterone-induced castration rats showed slight upregulation of caspase-3 and cleaved caspase-3, whereas cleistanthin A administration showed significant upregulation of these apoptotic markers (Figures 5E, 5F).

Cleistanthin B exerts cytotoxic and antiproliferative in testosterone-induced benign prostatic hyperplasia in castrated rats

**Figure 6:**
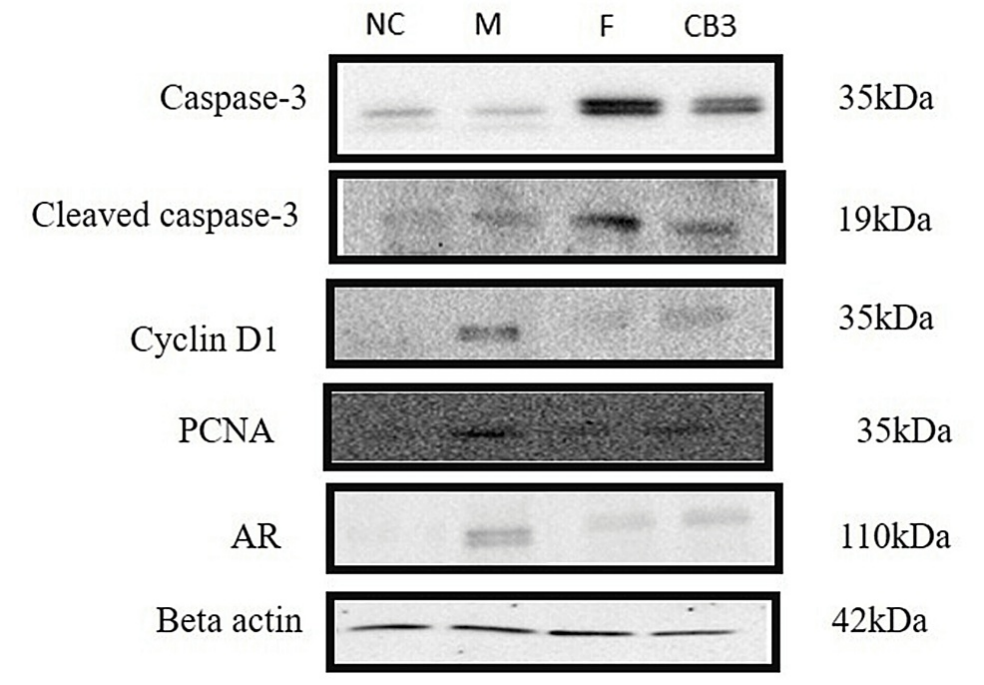
Western blotting for caspase-3, cleaved caspase-3, cyclin D1, PCNA, and AR protein expressions in the prostate tissue of negative control (NC), model (M), finasteride (F), and cleistanthin B (3 mg/kg body weight) groups. Apoptotic markers caspase-3 and cleaved caspase-3 protein expressions were downregulated in the BPH model group, and administration of cleistanthin B upregulated the expression of these apoptotic markers. β-actin was used as an internal control. Cyclin D1, PCNA, and AR expressions were elevated greatly in the testosterone-induced BPH model, while treatment with cleistanthin B at 3 mg/kg body hindered these elevations. PCNA = proliferating cell nuclear antigen; AR = androgen receptor; BPH = benign prostatic hyperplasia

To assess the cytotoxic effects of cleistanthin B in testosterone-induced BPH, we assessed the expression of apoptotic protein markers such as caspase-3 and cleaved caspase-3 by Western blotting (Figure 6).

**Figure 7:**
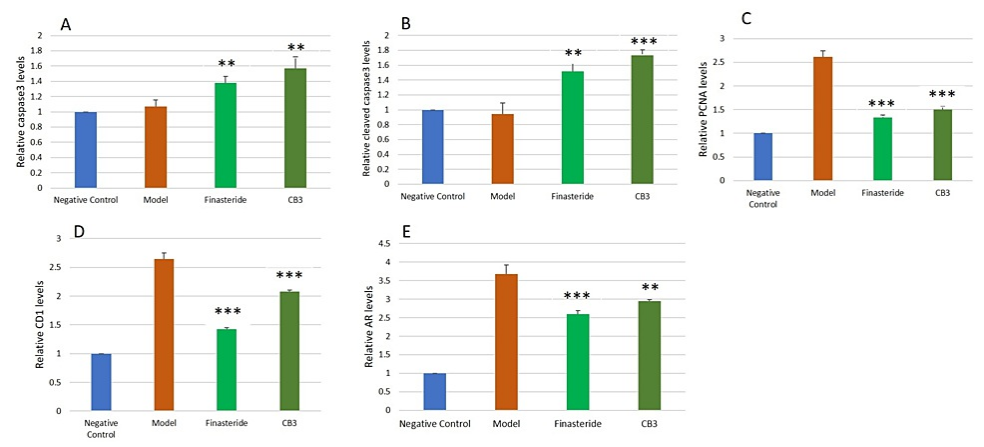
Relative caspase-3 (A), cleaved caspase-3 (B), PCNA (C), cyclin D1 (D), and AR (E) protein levels of the negative control, model, finasteride, and cleistanthin B at 3 mg/kg body weight (CB3). β-actin was used as an internal control. Densitometric analysis was performed using Image Lab 6.0.1 software, Bio-Rad Laboratories. Data are shown as mean ± SEM (n = 6). Statistical analysis was performed using ANOVA followed by the Tukey-Kramer multiple comparisons test. ** significant difference compared to the model group (p < 0.01). *** significant difference compared to the model group (p < 0.001). PCNA = proliferating cell nuclear antigen; AR = androgen receptor; SEM = standard error of the mean; ANOVA = analysis of variance

Treatment with cleistanthin B and finasteride upregulated the expression of these apoptotic proteins compared to the BPH model group (Figures 7A, 7B). In addition, the Western blot analysis revealed that cell proliferative markers PCNA and cyclin D1 levels were significantly elevated in the BPH group compared to the negative control group. Administration of cleistanthin B at 3 mg/kg body weight downregulated the expression of cell cycle proliferative markers cyclin D1 and PCNA (Figures 7C, 7D). On the other hand, prostatic tissue lysates of finasteride and cleistanthin B groups showed lowered expression of cyclin D1 and PCNA protein levels. Androgen receptor protein levels were found to be higher in the BPH model compared to the control group. Finasteride and cleistanthin B treatment groups showed a downregulated expression of androgen receptors (Figure 7E).

## Discussion

Effect of cleistanthins A and B on prostate weight, prostatic index, serum dihydrotestosterone levels, and the histomorphology in the testosterone-induced benign prostatic hyperplasia in castrated rats

In our study, the prostate weight and prostatic index were elevated in the testosterone-induced BPH in castrated rats compared to the negative control group. Treatment with cleistanthin A, cleistanthin B, and the comparator finasteride for 28 days in the BPH rat model ameliorated the increased prostate weight and prostatic index. These findings are corroborated by the serum DHT levels and the histopathological data of the prostatic tissues.

Oral administration of finasteride, cleistanthin A, and cleistanthin B decreased the serum DHT levels significantly in BPH rats compared to the model group. Androgens play an important role in the development and growth of reproductive organs. Prostatic cell differentiation and proliferation mainly depend on testosterone and the more potent DHT [22]. 5-α reductase is actively involved in the conversion of testosterone to DHT which has a higher affinity for androgen receptors. Both testosterone and DHT bind to androgen receptors and translocate to the nucleus and activate androgen-responsive genes which lead to the transcription of growth factors and ultimately prostatic hyperplasia [23,24]. The model group demonstrated prostatic hyperplasia which presents a tall columnar lining ductal epithelium with multilayering of the cells compared to the negative control group. Both investigational compounds cleistanthins A and B inhibited the deleterious effects of testosterone on the histoarchitecture of the prostate gland.

The macro findings supported by histopathological findings and hormonal levels give us the confidence to state that cleistanthins A and B are indeed effective against BPH in rats.

Effect of cleistanthins A and B on the serum pro-inflammatory cytokine levels in the testosterone-induced benign prostatic hyperplasia in castrated rats

Our study results suggest that testosterone induces an inflammatory response in prostatic tissue, as evidenced by the elevated levels of pro-inflammatory cytokines TNF-α and IL-1β. They even activate the transcription factors involved in cell growth [25]. In the present study, both cleistanthins A and B markedly decreased the serum pro-inflammatory cytokines TNF-α and IL-1β. Prostatic inflammation is commonly seen in BPH patients, and the histopathological investigation of the prostate tissues revealed the presence of inflammatory cells [26]. Hence, the fact that cleistanthins A and B reduce the serum DHT levels can also be a reason for the reduction in prostate weight.

Effect of cleistanthin A on IL-6/STAT-3/cyclin D1 signaling pathway in the testosterone-induced benign prostatic hyperplasia in castrated rats

Our study results reveal upregulation of the IL-6/STAT-3/cyclin D1 signaling pathway in the testosterone-induced BPH in castrated rats. Previous studies on the testosterone-induced rat BPH model have shown similar findings which led to the upregulation of the IL-6/STAT-3/cyclin D1 signaling pathway [27,28]. In BPH, the hyperproliferation of the prostatic epithelial and stromal cells and a decrease in apoptosis is observed. The increase in cell proliferation and chronic inflammation of the prostate activate the IL-6/STAT-3/cyclin D1 signaling pathway and play a significant role in the pathological progression of BPH and prostate cancer [29,30]. Chronic inflammation activates the IL-6 receptor, which, in turn, activates the JAK protein via GP130. Activation of JAK phosphorylates STAT-3 which translocates to the nucleus and initiates transcription of the genes involved in cell survival and proliferation, such as growth factors, angiogenic factors, and cytokines. Activation of STAT-3 leads to the upregulation of cyclin D1, BCL-2, and other related genes. STAT-3 is an oncogene involved in cell proliferation, chronic inflammation, and motility [31].

STAT-3 associates with V-ATPase in a coiled manner and regulates the activity of the V-ATPase which is important for the maintenance of the tumor microenvironment [32]. Cleistanthin A is a potent experimental anti-cancer V-ATPase inhibitor that has decreased the cell viability of various cancer cells [12,33]. Inhibition of V-ATPase by cleistanthin A may lead to the downregulation of the STAT-3-related signaling pathway. Western blotting results showed that administration of cleistanthin A decreased the expression of the IL-6/STAT-3/cyclin D1 signaling pathway significantly compared to the model group. Hyperproliferation of prostatic cells may also lead to the activation of the STAT-3 transcription molecule, which, in turn, promotes cellular growth. STAT-3 activation modulates the expression of the IL-6 gene in an autocrine manner via a positive feedback mechanism. STAT-3 binds to the promoter region of the IL-6 and upregulates the IL-6 expression [34,35].

Previous studies have demonstrated that the administration of testosterone in rats for the induction of BPH enhanced the proliferation of the prostatic tissue, which was supported by the upregulation of cell proliferative markers such as cyclin D1 and PCNA [36,37]. Hyperproliferation of stromal and epithelial cells of the prostate in BPH is involved in disease development and progression. Cell proliferation occurs in a sequential manner that comprises four phases, namely, G1, S, G2, and M, known as the cell cycle. The regulation of the cell cycle is critical and is dysregulated in various cancers. Cyclin D1 and PCNA are nuclear cell cycle regulatory proteins [38]. Cyclin D1 is necessary for the cell cycle progression in the G1 phase and is also involved at the beginning of the S phase [39]. PCNA acts as a cofactor for DNA polymerase, and increased expression of PCNA was observed during the transition of the G1 to the S phase of the cell cycle [40]. In this study, upregulation of both PCNA and cyclin D1 was observed in the rat BPH model. Administration of cleistanthins A and B for four weeks to BPH rats decreased the expression of these cell cycle regulatory markers significantly compared to the model group.

Effect of cleistanthins A and B on apoptosis in the testosterone-induced benign prostatic hyperplasia in castrated rats

Imbalance in cell proliferation and apoptosis play a significant role in the pathological development of BPH and prostate cancer [41]. The intrinsic and extrinsic pathways of cell death are executed by the caspase family of proteases. Both pathways finally activate executioner caspase-3 protease to carry out apoptosis. Subsequent activation of caspase-3 and cleaved caspase-3 leads to the proteolysis of cellular substrates and activates endonucleases involved in DNA fragmentation [42,43]. Caspase-3 and cleaved caspase-3 expression in BPH rat prostate tissues were only weakly detected. Cleistanthins A and B upregulated the protein expression of both caspase-3 and cleaved caspase-3 in BPH rats. These results suggest cleistanthins A and B induced apoptosis in BPH rats via activation of executioner caspase-3 and the subsequent caspase-dependent cell death pathway.

Proposed mechanism of action

Our study demonstrates significant evidence of apoptotic pathway in Western blot experiments with a prominent expression on the caspase pathway, with increased expression of caspase-3 and cleaved caspase 3 in the cleistanthin groups. We also demonstrated a marked decrease in the expression of PCNA in the same groups. The histomorphology demonstrated the absence of nucleoli and no evidence of mitotic activity in these groups, indicating a reduction in cell proliferation. Thus, we suggest that cleistanthins A and B act through multiple pathways with a reduction in cell proliferation and possibly an increase in programmed cell death.

The influence of cleistanthin B on the IL-6/STAT-3/cyclin D1 signaling pathway could not be studied but we do not expect it to be different from that of cleistanthin A because of the similarities in structure and actions between the compounds. Both compounds act by the downregulation of the IL-6/STAT-3/cyclin D1 signaling pathway leading to apoptosis and antiproliferative effects through cell cycle inhibition, as assessed by the cell proliferative markers cyclin D1 and PCNA. This might indicate the reduction in cell turnover and subsequent reduction in inflammation contributing to the overall effect of cleistanthins A and on BPH.

Cleistanthins A and B are α-adrenergic blockers that can influence the dynamic component in BPH. We are yet to study this aspect, and we expect the compounds would reduce the size of the prostate by virtue of their cytotoxic action. The fact that they reduce DHT levels similar to finasteride is a new, unexpected finding but we do not know if finasteride and cleistanthins share the same mechanism. We would like to hypothesize that cleistanthins inhibit 5-α reductase, the mechanism by which finasteride reduces the DHT levels.

Limitations of the study

The experimental BPH model does not exactly resemble a human pathological condition for BPH. We have proposed the probable mechanisms of cleistanthins A and B in the testosterone-induced BPH, but we could not provide any direct evidence by studying the ligand binding interactions.

## Conclusions

Cleistanthins A and B inhibit testosterone-induced BPH pathogenesis in castrated rats by exerting apoptosis, anti-proliferative effects, and possibly by reducing inflammation.
